# Phase-change behavior of RuSbTe thin film for photonic applications with amplitude-only modulation

**DOI:** 10.1038/s41598-024-59235-9

**Published:** 2024-04-17

**Authors:** Shogo Hatayama, Kotaro Makino, Yuta Saito

**Affiliations:** 1 Semiconductor Frontier Research Center, National Institute of Advanced Industrial Science and Technology (AIST), Tsukuba Central 2, Umezono 1-1-1, Tsukuba, 305-8568 Japan; 2 Research Center for Green X-Tech, Tohoku University, 6-6-11, Aoba-yama, Aoba-ku, Sendai, 980-8579 Japan; 3 Department of Materials Science, Graduate School of Engineering, Tohoku University, 6-6-11, Aoba-yama, Aoba-ku, Sendai, 980-8579 Japan

**Keywords:** Phase change material, High thermal stability, Optical properties, Transition metal, RuSbTe, Materials for optics, Materials for devices

## Abstract

Ge_2_Sb_2_Te_5_ (GST), the most mature phase-change materials (PCM), functions as a recoding layer in nonvolatile memory and optical discs by contrasting the physical properties upon phase transition between amorphous and crystalline phases. However, GST faces challenges such as a large extinction coefficient (*k*) and low thermal stability of the amorphous phase. In this study, we introduce RuSbTe as a new PCM to address the GST concerns. Notably, the crystallization temperature of the amorphous RuSbTe is approximately 350 °C, significantly higher than GST. A one-order-of-magnitude increase in the resistivity contrast was observed upon phase transition. The crystalline (0.35–0.50 eV) and amorphous (0.26–0.37 eV) phases exhibit relatively small band gap values, resulting in substantial *k*. Although RuSbTe demonstrates a *k* difference of approximately 1 upon crystallization at the telecommunications C-band, the refractive index (*n*) difference is negligible. Unlike GST, which induces both phase retardation and amplitude modulation in its optical switch device, RuSbTe exhibits amplitude-only modulation. This study suggests that RuSbTe has the potential to enable new photonic computing devices that can independently control the phase and amplitude. Combining RuSbTe with phase-only modulators could open avenues for advanced applications.

## Introduction

Phase-change materials (PCMs) exhibit a reversible phase transition between the amorphous and crystalline phases^1^. Generally, the amorphous phase exhibits high electrical resistivity and low optical reflectance, whereas the crystalline phase exhibits low resistivity and high reflectance. The contrast in the physical properties upon phase change enables PCM to be used for nonvolatile storage applications such as nonvolatile memory and optical discs^1^. In addition to these storage techniques, recent studies have demonstrated that PCMs are promising for rewritable photonic elements used in reconfigurable photonic devices^2–4^.

Owing to the successful demonstration of nonvolatile memory and optical disc applications, the Ge_2_Sb_2_Te_5_ (GST) is the most mature and promising PCM for photonic applications with a large contrast in physical properties upon phase transition and high state discrimination^1^. The large shift in the refractive index (*n*) of 3.56 at a wavelength of 1550 nm, which corresponds to the telecommunications C-band, provides distinct states in transmission, enabling photonic computing^5,6^. Meanwhile, the extinction coefficient (*k*) of GST limits the device size and scalability. This emphasizes the importance of developing low-loss PCMs such as Sb_2_S_3_ or Sb_2_Se_3_^3^. Another concern regarding GST is the low thermal stability of the amorphous phase, which must be addressed. Long-term retention of encoded data is required for computing applications, such as synaptic weight in neuromorphic computing. To this end, the relatively low crystallization temperature *T*_x_ of GST (150 °C) should be improved. Transition-metal-included PCMs are one of the solutions for realizing long data retention owing to their high thermal stability^7–11^.

In the Ru–Sb–Te ternary system, the stoichiometric compound RuSbTe exhibits semiconductor characteristics^12^, and it is theoretically predicted that *k* decreases with increasing wavelength in the infrared region^13^. Although an amorphous phase of RuSbTe has not been demonstrated to date, the melting point of the bulk materials has been reported in the temperature range of 700–900 °C^12^, comparable to conventional PCMs^14,15^. In fact, the as-deposited RuSbTe film shows an amorphous phase, as demonstrated in the following section. Moreover, because Ru doping significantly enhances the thermal stability of amorphous Sb_2_Te, and GeTe^10,11^, a high *T*_x_ is expected for amorphous RuSbTe. Based on this background, we aimed to develop a stoichiometric ternary RuSbTe material with high thermal stability and optical property modulation upon phase changes for photonic applications.

## Results and discussion

**Figure 1:**
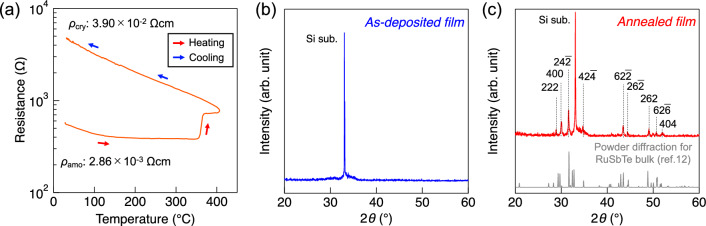
(**a**) Temperature dependence of resistance for the as-deposited film. The XRD patterns for as-deposited film (**b**) and 400 °C-annealed film (**c**). Bragg reflection depicted as a gray-colored line indicates a powder pattern for RuSbTe crystal structure simulated from crystal structure parameters: *a* = 6.56 Å, *b* = 6.61 Å, *c* = 6.64 Å, and *β* = 113.6° (sd_1820542 in Springer Material online database^31^).

Figure 1a shows the temperature dependence of the resistance of the as-deposited film. During the heating process, the resistance gradually decreases up to 100 °C. The resistance remains nearly constant within 100–300 °C but suddenly increases at 350 °C. During cooling, a highly resistive state was maintained, followed by an increase in the resistance to room temperature. In the X-ray diffraction (XRD) pattern of the as-deposited film, there was no distinct Bragg reflection except for that of the Si substrate, indicating an amorphous phase (Fig. 1b). Meanwhile, the XRD pattern for the 400 °C-annealed film shows Bragg reflection corresponding to the RuSbTe crystal structure (Fig. 1c). By analyzing the XRD pattern for the annealed film by Williamson-Hall method, the grain size was determined to be 2.98 nm, indicating polycrystalline microstructure with small grains. The resistivity for the crystalline phase (3.90 × 10^–2^ Ωcm) was found to be an order of magnitude larger than that for the amorphous phase (2.86 × 10^–3^ Ωcm). This type of resistive change classifies RuSbTe as an inverse resistance change PCM^8,16–18^. Since the sudden increase in resistance corresponds to the crystallization in inverse resistance change PCMs^8,18^, the *T*_x_ can be approximately 350 °C, which is much higher than GST by 200 °C. Such high thermal stability is assumed to originate from the high number of coordination bonds formed in the local structure around Ru in the amorphous phase, as predicted for Ru-doped GeTe^11^.

**Figure 2:**
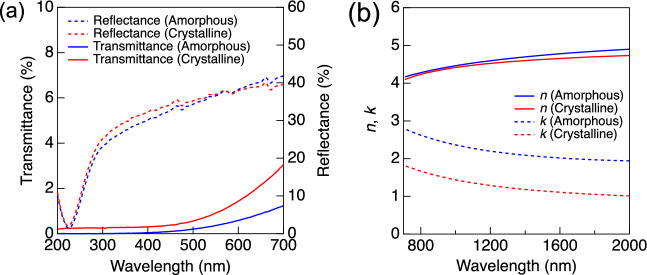
(**a**) Transmittance (solid lines) and reflectance (dashed lines) spectra for the amorphous and crystalline RuSbTe films. (**b**) Refractive index (solid lines) and extinction coefficient (dashed lines) for the RuSbTe films.

The optical contrast upon phase transition is a unique feature of PCM. The reflectance change upon phase transition was used to record the principle of optical discs^1^. GST shows a 20–30% contrast in reflectance at 630 nm upon phase transition, which is used for data reading in product^1^. In contrast to the optical characteristics of GST, the reflectance spectra of RuSbTe were nearly the same for the two phases in the visible region, as shown in Fig. 2a. The increase in reflectance across the wavelength range of 300–700 nm is attributed to the gradual escalation of *n* in the infrared region, as illustrated subsequently (Fig. 2b), preceded by a similar trend observed within the visible range. The transmittance gradually increases with increasing wavelength, and the crystalline phase exhibits higher values than the amorphous phase in this measurement range (Fig. 2a).

These results indicated that the optical properties of RuSbTe differed from those of conventional PCMs. ﻿As mentioned in Introduction, the refractive index and extinction coefficient in the infrared region are more important for photonic computing devices than the optical properties in the visible region. Figure 2b shows *n* and *k* for films in the infrared range (700–2000 nm). The Tauc–Lorentz model was used for data fitting. Similar to the reflectance, the *n* spectra do not show a clear difference, providing the contrast in the *n* at 1550 nm, Δ*n*, of 0.12. Compared to the Δ*n* for PCMs considered for photonic computing devices such as GST (3.56), Sb_2_S_3_ (0.60), and Sb_2_Se_3_ (0.77)^3,6^, the RuSbTe showed negligible contrast in the refractive index. In contrast to *n*, the *k* shows a clear difference between the two phases. Both phases showed *k* values greater than 1 in the infrared range owing to their small band gaps, as discussed later.

**Figure 3:**
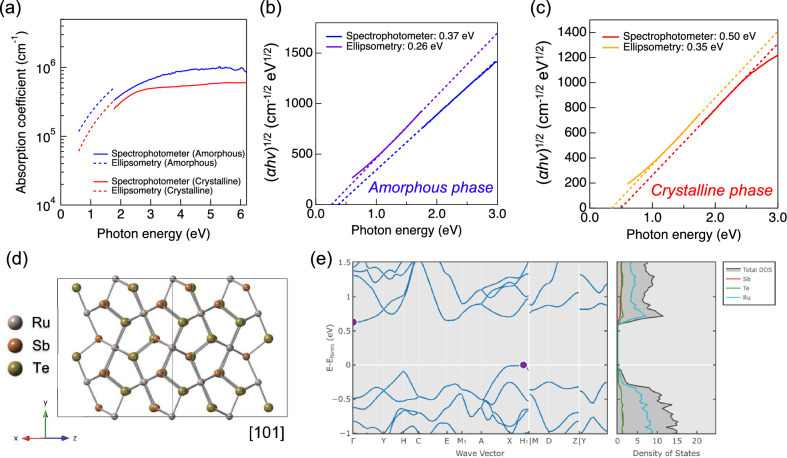
(**a**) Absorption coefficient for RuSbTe films. Red and blue lines indicate crystalline and amorphous phases, respectively. Dashed and solid lines indicate the absorption coefficient spectra obtained based on ellipsometry and spectrophotometer, respectively. (**b,c**) show Tauc plots assuming an indirect transition for amorphous and crystalline phases, respectively. Purple and yellow solid lines are calculated based on the absorption coefficient obtained by ellipsometry. Blue and red solid lines are calculated based on the absorption coefficient obtained by the spectrophotometer. Dashed lines are the linear fitted curves. (**d**) Crystal structure for RuSbTe viewed from [101] direction. (**e**) Band structure for crystalline RuSbTe calculated by density functional theory calculation. This figure is reprinted from Materials Project (mp-1102857)^24,25^.

The absorption coefficient, *α*, in the visible and infrared ranges was calculated using the following equations^19–21^:

1 $$\alpha =\frac{1}{d}{\text{ln}}\left(\frac{{\left(1-R\right)}^{2}+{\left({\left(1-R\right)}^{4}+4{R}^{2}{T}^{2}\right)}^\frac{1}{2}}{2T}\right),$$

2 $$\alpha =\frac{4\pi k}{\lambda },$$

where *d* is the thickness, *R* is the reflectance, *T* is the transmittance, and *λ* is the wavelength. The *α* for visible range was calculated based on Eq. (1) using the obtained results by spectrophotometer measurement, and that for infrared range was calculated based on Eq. (2) using the obtained results by ellipsometry measurement. Figure 3a shows the calculated *α* spectra. In both amorphous and crystalline phases, the calculated *α* based on the ellipsometry results is larger than that based on the spectrophotometer results. However, there is a tendency for *α* to gradually decrease in the visible region, followed by a drastic decrease in the infrared region. The *α* is expressed with considering the Fresnel reflection and scattering is expressed as followings^22^:

3 $$\alpha =\frac{{\text{ln}}(1-{R}_{1})(1-{R}_{2})(1-{R}_{3})(1-S)}{d}-\frac{{\text{ln}}T}{d},$$

where *S* is the assumed fraction of light that does not reach the detector because of scattering and *R*_*i*_ (*i*: 1, 2, 3) is the Fresnel reflection at the interfaces of air/film (*R*_1_), film/substrate (*R*_2_), and substrate/air (*R*_3_). The *α* value calculated without accounting for Fresnel reflection and scattering is underestimated, as indicated by the positive sign of the first term on the right-hand side of Eq. (3). Equation (1) does not include the impact of Fresnel reflection and scattering, resulting in a lower *α* derived from spectrophotometer measurements compared to that obtained from ellipsometry measurements. Tauc plots were performed to determine the bandgap (*E*_g_), as shown in Fig. 3b,c. In addition to the amorphous phase^23^, an indirect transition was assumed in the Tauc plots for the crystalline phase because RuSbTe with P21/c symmetry (Fig. 3d) was predicted to show an indirect transition by density functional theory (DFT) calculations (Fig. 3e)^24,25^. From these Tauc plots, the *E*_g_ was determined to be 0.26–0.37 eV and 0.35–0.50 eV for the amorphous and crystalline phases, respectively. For both phases, the *E*_g_ estimated from the spectrophotometer measurement is higher than that estimated from the ellipsometry measurement due to the difference in the *α*. The *E*_g_ for the RuSbTe films is smaller values than the photon energy in infrared range (0.62–1.77 eV), leading to the large *k*. Furthermore, the non-zero values of *E*_g_ observed for both the amorphous and crystalline phases contribute to increase the transmittance as the wavelength increases (Fig. 2a).

**Figure 4:**
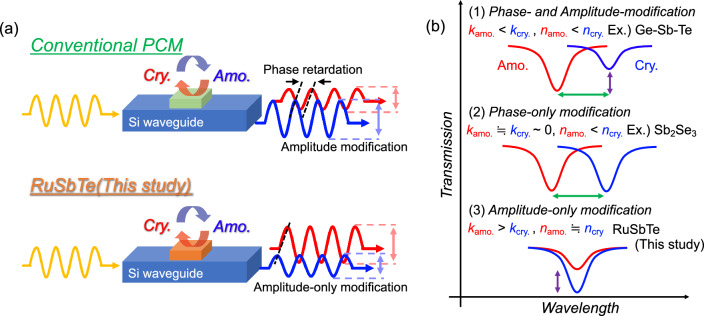
(**a**) Schematics for PCM-based programable photonic devices fabricated with conventional PCM (upper side) and RuSbTe (lower side). (**b**) Schematics for transmission change of PCM-embedded optical ring resonator upon phase transition in the cases of conventional PCM (1), phase-only modification (2), and amplitude-only modification (3).

Contrary to the aim mentioned in the Introduction, the RuSbTe films showed large absorption in both phases. However, it was found that RuSbTe showed a different response in the refractive index upon phase change from conventional PCMs, suggesting the possibility of realizing a new type of device. The changes in *n* and *k* obtained in conventional PCMs cause both phase retardation and amplitude modulation, which can be utilized for phase-shifter-type optical switches by combining a Mach–Zehnder interferometer and simple transmission modulator with a straight waveguide, respectively. For a transmission modulator, a phase shift is not favorable because unexpected signal modulations can be induced. RuSbTe exhibits a large contrast in *k* with nearly no change in *n*, leading to an amplitude-only modification, as shown in Fig. 4a.

Figure 4b schematically depicts the transmission changes in PCM-embedded optical ring resonator. In the case of conventional PCMs such as GST, both the transmission and wavelength for minimum transmission change upon phase transition, as shown in (1) of Fig. 4b, which is not straightforward for optical controlling. Meanwhile, recent studies demonstrated phase-only modulator using Sb_2_Se_3_, Ge_2_Sb_2_Se_4_Te, and Ge_2_Sb_2_Te_3_S_2_^26–28^, leading to a phase shift remaining in the transmission ((2) of Fig. 4b). Because the transmission can be simply modified, RuSbTe can become a component to realize new photonic computing devices, which independently control the phase and amplitude, by combining phase-only modulators.

In all-optical switches, metallic heaters cannot be utilized, necessitating the induction of phase change through laser irradiation. However, employing a low-absorption PCM in a phase-only modulator requires high optical power for switching, potentially causing a damage to surrounding components within the optical circuit. To mitigate heat propagation induced during phase-only modulator switching, PCM must be adequately isolated from components, resulting in a large circuit size. Conversely, RuSbTe demonstrates efficient laser absorption owing to its high *k*. Consequently, RuSbTe offers an advantage in reducing the size of the all-optical switch circuit.

In summary, the phase-change behavior of RuSbTe films was investigated. The fabricated films exhibited an inverse change in resistance between the low-resistive amorphous and highly-resistive crystalline phases. The crystallization temperature of the film was much higher than that of the GST, indicating its high thermal stability. The small optical contrasts in the reflectance and transmittance ruled out the possibility of using RuSbTe films as traditional optical discs. However, the significant difference in *k*, while almost no difference in *n*, indicates that the RuSbTe film is a promising reconfigurable transmission modulator with amplitude-only modulation.

## Experimental methods

RuSbTe films were deposited on SiO_2_ (100 nm)/Si or glass substrates by radiofrequency magnetron sputtering using a pure Ru metal target and an Sb_2_Te_3_ alloy target. Because the composition of the deposited film tends to be different from that of the original target owing to the different sputtering yields and ion energies of different elements^29^, a slightly Te-rich alloy composition was employed as a counterpart for Ru. The films were fabricated with a thickness of 100 nm, followed by surface coating with a 10 nm-thick ZrO_2_ layer without breaking the vacuum. The film thickness was measured using a stylus profilometer (DektakXT; Bruker). The composition of the film was Ru_0.31_Sb_0.28_Te_0.41_ by X-ray fluorescence measurement (EA6000VX; HITACHI).

The temperature dependence of the resistance of the as-deposited film was measured in an Ar atmosphere at atmospheric pressure. The resistance was measured using a resistance-measure unit (MILA-5000 and MILA-5000-TER; ADVANCE RIKO, Inc.) during annealing up to 400 °C within 10 min. This was followed by cooling to room temperature in a lamp furnace. X-ray diffraction (XRD) measurements were performed on the RuSbTe film grown on a SiO_2_ (100 nm)/Si substrate with a Cu Kα source (λ = 0.1542 nm) using a conventional 2θ/θ geometry.

The reflectance (*R*) and transmittance (*T*) of 100-nm-thick RuSbTe films grown on glass substrates were measured for the perpendicular to the film surface in the wavelength range 200–700 nm using a spectrophotometer (Solid-Spec 3700i; SHIMADZU). The reflectance was measured relative to that of the Al mirror. The transmittance was measured relative to that of the glass substrate. The obtained results using spectrophotometer were analyzed without scattering.

The complex indices of the refraction spectra of the RuSbTe films were measured using a spectroscopic ellipsometer (UVISEL 2; HORIBA). Measurements were performed in the spectral range of 750–2000 nm at an incident angle of 70°. The Tauc–Lorentz model^30^ was used for data analysis.

## Data Availability

All data are available in the main text.
